# A comparison of the effects of direct oral anticoagulants versus vitamin K antagonists and antiplatelet agents on the timing and outcomes of hip fracture surgery in patients older than 65 years: the ORTHO-GER-DOAC study

**DOI:** 10.1007/s41999-025-01198-9

**Published:** 2025-04-23

**Authors:** Emilio Martini, Rossella Tozzi, Giulia Annessi, Laura Borgese, Maria Lia Lunardelli, Calogero Alfonso, Caternicchia Filippo, Elvira Grandone, Benilde Cosmi

**Affiliations:** 1 Geriatric and Orthogeriatric Unit, Azienda Ospedaliero Universitaria Modena, Ospedale Civile Baggiovara, Modena, Italy; 2 Angiology and Blood Coagulation Unit, Department of Medical and Surgical Sciences, University of Bologna, IRCSS Azienda Ospedaliera-Universitaria di Bologna, Via P. Albertoni, 15, Bologna, Italy; 3 Geriatric Acute Care, Orthogeriatric Unit and Center for Diagnosis of Cognitive Disorders and Dementia, IRCCS Azienda Ospedaliera-Universitaria di Bologna, Bologna, Italy; 4 Orthopedics Unit, Ospedale S.Maria Bianca, Mirandola, Italy; 5 Department of Orthopedics and Traumatology, IRCSS Azienda Ospedaliera-Universitaria di Bologna, Bologna, Italy; 6 Thrombosis and Haemostasis Unit Research Institute “Casa Sollievo della Sofferenza”, San Giovanni Rotondo, Foggia Italy; 7 Angiology and Blood Coagulation Unit, Department of Medical and Surgical Sciences, University of Bologna, Bologna, Italy

**Keywords:** Hip fracture, Direct oral anticoagulants, Vitamin K antagonists, Antiplatelet agents, Bleeding

## Abstract

**Introduction:**

The use of direct oral anticoagulants (DOACs) may delay surgery in older hip fracture patients.

**Aim:**

To assess whether preoperative DOAC activity measurement enables surgery within 48 h in hip fracture patients at a similar prevalence compared to patients receiving other antithrombotics or no antithrombotics.

**Methods:**

A retrospective observational cohort study of hip fracture patients older than 65 years admitted to three Orthogeriatrics units in Italy from 2015 to 2022 was conducted. At admission, demographical and comorbid conditions were recorded, and antithrombotics were stopped. Patients on vitamin K antagonists (VKAs) underwent international normalized ratio (INR) assessments and received vitamin K to achieve an INR below 1.5. Patients receiving DOACs who were enrolled before 2018 underwent daily drug testing, and surgery was performed only after DOAC levels were near or below trough levels. Hours from hospital admission to surgery, perioperative total blood loss, major bleeding and mortality at 90 days were recorded.

**Results:**

Amongst the 747 patients (median age 85 years; M/F: 192/555), the prevalence of surgery within 48 h was significantly lower amongst patients receiving DOACs (47%) than amongst patients receiving antiplatelet agents (77%) and patients receiving no antithrombotic agents (73%). Preoperative DOAC measurements significantly delayed the time to surgery (median 51 vs. 42 h: *P* < 0.05). The major bleeding and mortality rates at 90 days did not differ based on the type of antithrombotics used. The degree of perioperative blood loss was greater in patients receiving DOACs, regardless of drug measurement, than in patients taking other antithrombotics.

**Conclusions:**

DOAC measurement may delay hip fracture surgery, as even low presurgical levels of DOACs are associated with greater perioperative blood loss.

**Supplementary Information:**

The online version contains supplementary material available at 10.1007/s41999-025-01198-9.

## Key summary points

**Aim:**

To assess whether preoperative DOAC activity measurement enables surgery within 48 h amongst hip fracture patients at a similar prevalence compared with patients receiving other antithrombotics and patients receiving no antithrombotics.

**Findings:**

Preoperative DOAC drug measurement significantly delayed the time to surgery, whereas DOACs were associated with greater perioperative blood loss, even amongst patients with low presurgical drug levels and regardless of the type of anaesthesia.

**Message:**

In patients with hip fracture who are receiving DOACs, a protocol for expedited surgery can be applied in which DOACs are stopped before surgery for at least 48 h for factor Xa inhibitors and according to renal function and up to 96 h after the last dose of dabigatran. In this protocol, no drug-level testing is performed.

**Supplementary Information:**

The online version contains supplementary material available at 10.1007/s41999-025-01198-9.

## Introduction

Proximal femur fractures (hip fractures) are increasingly common in developed nations as well as in developing nations due to the rapid ageing of the global population [1]. When hip fractures occur, the aim is to treat the injury surgically, reduce pain and reduce the morbidity associated with being bed bound, thus allowing for early mobilisation and discharge from the hospital [2].

The management of hip fracture is subject to surveillance at the national level in Italy. The best practise in hip fracture management is specified in the National Outcome Registry (Piano Nazionale Esiti), which monitors surgery within 48 h after admission [3]. Numerous studies have associated surgical delay with increased length of stay, major morbidity and increased mortality rate (up to 9.4%) [1–12]. Therefore, surgery within 48 h after admission for patients older than 65 years for hip fracture is a hospital benchmark because of its inverse relationship with mortality.

The use of anticoagulants, especially for atrial fibrillation (AF), is increasing amongst elderly individuals. A retrospective analysis of 15,099 subjects with proximal femur fractures from January 2016 to December 2018 in Germany from the Registry for Geriatric Trauma (ATR-DGU) revealed that 11% (n = 1595) of patients took direct oral anticoagulants (DOACs) at the time of fracture, whereas 9.2% (n = 1325) were receiving vitamin K antagonists (VKAs) [13]. During the study period, there was a shift from VKAs to DOACs. The time to surgery of patients on DOACs and patients on VKAs was longer than that of patients who did not receive any anticoagulants. No significant differences in complications, type of anesthesia or mortality were found between patients receiving DOACs and those receiving VKAs [13]. The proportion of subjects with hip fracture on anticoagulants was similar to that reported in other countries [14, 15].

Many scientific societies have published guidelines regarding the management of DOACs at the time of anesthesia and surgery [16–24]. However, these guidelines do not consider all the perioperative aspects of hip fracture treatment or are not specific to hip fracture [25]. In subjects receiving DOACs, clinical practise guidelines previously suggested waiting at least 48 h after stopping factor Xa inhibitors and up to 96 h after stopping dabigatran before performing surgery due to a high risk of bleeding; drug testing was not used, but renal function was measured and the weaning of DOAC anticoagulant effects were predicted [24]. However, no evidence is available regarding any changes in the duration of perioperative DOAC interruption based on residual anticoagulant levels. In addittion, safe plasma levels of DOACs for different procedures have yet to be determined [25]. No randomised clinical trials have been performed to compare the effects of DOAC measurements with no measurements on outcomes such as time to surgery, bleeding complications or mortality [26]. More recently, the European Society of Cardiology guidelines suggested that, in the case of time-sensitive noncardiac surgery, DOACs could be stopped at least 12 h prior to surgery [24]. However, for interventions with a very high risk of bleeding, such as those involving spinal or epidural anesthesia, interruption of DOACs for up to five half-lives and reinitiation after 24 h should be considered (class of recommendation IIA, level of evidence: C) [24].

The aim of this study was to compare the time to surgery with the preoperative DOAC measurement with the time to surgery after an expedited surgical protocol without preoperative DOAC measurements in patients older than 65 years who underwent hip fracture surgery.

## Materials and methods

### Experimental design

A retrospective observational cohort study was conducted from 2015–2022 amongst hip fracture patients older than 65 years who were admitted to three Italian centres: the Orthogeriatric Unit, IRCCS Azienda Ospedaliero-Universitaria di Bologna; the Orthopedic Unit Opera Pia Sollievo della Sofferenza, S. Giovanni Rotondo, Foggia, Italy; and Baggiovara Hospital, Modena, Italy.

The local ethics committees approved the study, and patient informed consent was waived due to the retrospective design of this study.

The inclusion criteria were as follows: age above 65 years, hip fracture occurring less than 24 h before admission, and being a candidate for osteosynthesis or hip prosthesis. The exclusion criteria were as follows: age younger than 65 years, nonproximal hip fracture or pathological fracture, ongoing treatment with heparin or low-molecular-weight heparin and hip fracture occurring more than 24 h before admission.

At admission, demographical information, type of hip fracture, and comorbid conditions were collected along with the Cumulative Illness Rating Scale (CIRS). The CIRS scale is a comorbidity index consisting of 14 items exploring impairment of different organs and systems. Each item is rated from 1 (no impairment) to 5 (extremely severe impairment) [27]. The CIRS Severity Index (CIRS-SI) was calculated as the average of all the CIRS items, and the CIRS comorbidity index (CIRS-CI) was based on the number of organ systems with moderate to more significant impairment (score ≥ 3) [28, 29].

The Barthel Index is used to assess the level of functional autonomy, especially from a motor point of view. The score varies from 0 to 100, where the highest score is an indicator of complete independence [30].

The Activity of Daily Living Katz Index (ADL) is used to evaluate the prefracture functional status in basic activities of daily living. The six-item Katz index assesses with a unique yes/no question the performance in the functions of bathing, dressing, toileting, transferring, continence and feeding. A score of 6 indicates full function, 4 indicates moderate impairment, and 2 or less indicates severe functional impairment [31].

Mental functioning status was assessed with the Short Portable Mental Status Questionnaire (SPMSQ), which consists of 10 questions, where poor performance (a greater number of incorrect answers) is correlated with the presence of cognitive disorders [32].

Routine laboratory tests were also performed. All ongoing antithrombotic agents were stopped except for antiplatelet agents. An expedited surgical perioperative protocol involving anticoagulant reversal was applied to expedite surgery within 48 h of admission. Patients taking VKA underwent INR testing and received vitamin K (5 mg intravenously) on the day of admission, and the INR was repeated the next day or preoperatively to obtain a value < 1.5.

Patients receiving DOACs underwent daily drug testing from 2015 to 2018, and surgery was performed only after DOAC levels were near or below trough levels in the centre of Bologna [16, 33]. DOAC levels were measured according to local laboratory methods for factor Xa inhibitors or a factor IIa inhibitor (dabigatran). After 2018, a new protocol was applied, in which DOACs were stopped before surgery for at least 48 h for factor Xa inhibitors and, according to renal function, for up to 96 h for dabigatran since the last dose without any drug-level testing. The type of anaesthesia (general or spinal) was also recorded, as was the length of hospital stay in the Orthopaedics Department.

### Outcomes and follow-up

The time (hours) from hospital admission to surgery was recorded before and after the application of the new protocol for DOAC interruption. The time to surgery amongst patients receiving DOACs, either with or without preoperative drug testing, was compared with that of patients receiving either VKAs or antiplatelet agents or no antithrombotic drugs. The secondary outcomes were total blood loss, major and clinically relevant bleeding and mortality at 90 days. Total blood loss was calculated according to haemoglobin levels before and after surgery (on the fourth postoperative day), the number of transfused red blood cells, sex, weight and height according to Good et al. [34].

Major bleeding complications were recorded during the hospital stay and within 30 days. These events were adjudicated by local investigators according to the International Association of Thrombosis and Haemostasis (ISTH) Scientific Committee, and they included haemorrhage from the gastrointestinal, urinary and cerebrovascular systems [35]. All-cause mortality was assessed at 90 days. Data from the follow-up period were obtained by checking medical electronic records and by contacting the patients or the patient’s general practitioners by phone if information was not available in the electronic database. Patients were considered lost to follow-up whenever they did not answer five telephone calls on 5 different days at different hours and their family physicians did not have direct news about them.

### Statistical analysis

Categorical variables are expressed as frequencies and percentages; continuous variables are expressed as the means, medians and interquartile ranges (IQRs). A nonparametric test (Mann‒Whitney U test) was used for comparisons between continuous variables, and the Chi‒square test or Fisher’s exact test was used for comparisons of categorical variables. Multivariable logistic regression analysis was conducted to explore the independent effects of variables that were significantly associated with the time to surgery (< 48 h vs. > 48 h) in the univariable analysis to avoid overfitting.

A value of *P* < 0.05 was considered to indicate statistical significance. Statistical analysis was carried out using the SPSS statistical software package (version 23; SPSS Inc. Chicago, Illinois, USA).

## Results

Table 1 shows the characteristics of the 747 included patients, of whom 421 were recruited from IRCSS Azienda Ospedaliero-Universitaria di Bologna, 46 were recruited from Ospedale Sollievo della Sofferenza, S.Giovanni Rotondo, Foggia, and 280 were recruited from Baggiovara Hospital, Modena.

**Table 1:** Characteristics of the enrolled patients

Gender	555 (74.3%) females 192 (25.7%) males	Available in
Age (median; IQR)	85 (81–89)	747
Severity Index (CIRS-S; median IQR)	1.76 (1.38–2.00)	502 (67%)
Comorbidity Index (CIRS-C) (median; IQR)	3 (2–4)	504 (67%)
ADL (median; IQR)	5 (3–6)	625 (84%)
ADL 6/6: n. (% fully independent)	333 (45)	747
Barthel Index (median; IQR)	90 (60–100)	649 (87%)
Barthel Index 100/100: n. (% fully independent)	269 (36)	747
SPMSQ (median; IQR)	3 (1–6)	460 (61%)
SPMSQ > 4 (moderate/severe cognitive impairment) *n* (%)	432 (58)	
ASA score (median; IQR)	3 (3–3)	504 (67%)
Fracture type, *n* (%)	Intracapsular 308 (41.2%) Extracapsular 439 (58.8%)	
Surgical procedure, *n* (%)	Total arthroplasty 22 (2.9%) Hemiarthroplasty 256 (34.3%) Osteosynthesis with intramedullary nail 439 (58.8%) Osteosynthesis with cannulated compression screw 30 (4%)	
Type of anaesthesia (general/neuraxial) *n* (%)	187/560 (25%/75%)	
Time to surgery (median; IQR)	45 (27.1–52.1)	
Number of patients undergoing surgery within 48 h from admission	485 (67.6%)	

ADL, Activities of Daily Living Katz Index; ASA, American Society of Anaesthesiology; IQR, interquartile range; CIRS, Cumulative Illness Rating Score; SPMSQ, Short Portable Mental Status Questionnaire

A total of 74.3% of the patients were females, and the median age was 85 years (IQR: 81–89). Patients had high comorbidity indices, a high incidence of cognitive impairment and a low incidence of total functional capacity before hip fracture. The American Society of Anesthesiology (ASA) score was high (median: 3), and only 25.6% of patients had a low surgical risk (ASA score of 1 or 2).

Most hip fractures were extracapsular (58.8%), and most patients underwent osteosynthesis with an intramedullary nail (58.8%) (Table 2). Neuraxial anaesthesia was employed in most patients (75%) and in all patients in whom preoperative DOAC testing was performed.

**Table 2:** Time to surgery, percentage of patients who underwent surgery within 48 h according to the type of anticoagulant used and length of stay (LOS) in the orthopaedic wards

Type of antithrombotic drug	None	Antiplatelet agents	Vitamin K antagonists	Direct oral anticoagulants	*P*
Number of patients (%)	274 (37)	249 (33)	76 (10)	148 (20)	
Time to surgery hours (mean, SD)	46.5 (92.1)	42.7 (30.6)	64.8 (44.5)	64.9 (44.5)	0.003
Time to surgery hours (median, IQR)	41.8 (24.5–49.3)	42.0 (25.1–48.1)			0.82
	41.8 (24.5–49.3)		48.55 (42.6–68.7)		0.01
	41.8 (24.5–49.3)			50.7 (42.4–72.1)	0.00
Surgery within 48 h Number of patients (%)	200 (73)	192 (77)	40 (53)	70 (47)	0.00
LOS (median; IQR)	10 (8–13)	9 (7–12)			0.56
	10 (8–13)		9.5 (8–12.25)		0.99
	10 (8–13)			13 (10–14)	0.00
	10 (7–12.2)			13 (10–14)	0.00

IQR, interquartile range

Table 2 shows the time to surgery and percentage of patients who underwent surgery within 48 h stratified by the type of anticoagulant. The majority of patients (73%) were taking antithrombotic drugs before admission, especially antiplatelet agents (either acetylsalicylic acid or thienopyridines—33%). Amongst these patients, only 47% of those receiving DOACs underwent surgery within 48 h after admission, which was a significantly lower proportion than that amongst patients receiving antiplatelet agents (77%) or no antithrombotics (73%) but similar to the proportion of patients receiving VKAs who underwent surgery withing 48 h after admission (53%). The length of stay in the orthogeriatric/orthopaedics unit was significantly longer for patients taking DOACs than for patients receiving other antithrombotic agents or no antithrombotic agent.

Tables 3 and 4 show the time to surgery amongst patients who underwent preoperative DOAC measurements (n = 34; 23%) compared with patients who did not undergo preoperative DOAC measurements (n = 114 in all 3 centres and 12 in Bologna). Preoperative DOAC measurements delayed the time to surgery (76.3 h vs. 61.2, *P* = 0.09 overall, and 78 h vs. 61 in Bologna), although this difference was not significant in the limited sample.

**Table 3:** Comparison of the timing of surgery (hours) and the number of patients who underwent surgery within 48 h either with or without preoperative direct oral anticoagulant (DOAC) measurements

	Timing of surgery with preoperative DOAC measurement (*n* = 34)	Timing of surgery without preoperative DOAC measurement (*n* = 114)	*P*
Mean (SD)	76.3 (64.4)	61.25 (35.4)	0.09
Median (IQR)	50.9 (46.6–82.0)	50.4 (42.1–71.3)	0.84
Surgery within 48 h (%)	47.0%	47.1%	0.99

IQR, interquartile range

**Table 4:** Comparison of the timing of surgery (hours) and number of patients who underwent surgery within 48 h either with or without preoperative direct oral anticoagulant (DOAC) measurements at the centre in Bologna

	Timing of surgery with preoperative DOAC measurement (*n* = 34)	Timing of surgery without preoperative DOAC measurement (*n* = 12)	*P*
Mean (SD)	77.8 (66.1)	61.25 (75.4)	0.46
Median (IQR)	50.9 (46.6–82.0)	50.4 (42.1–71.3)	0.98
Surgery within 48 h (%)	47.0%	52.1%	0.89

IQR, interquartile range

In the initial part of the study, from 2015–2018, when the frequency of hip fracture patients receiving DOACs slowly increased, preoperative anticoagulant activity measurements were performed in all patients taking DOACs in Bologna, where all patients received neuraxial anaesthesia. After 2018, the new protocol was implemented, and preoperative DOAC measurements were stopped to allow expedited surgery.

No difference in the time to surgery was detected between patients receiving neuraxial anaesthesia and those receiving general anaesthesia (54.11 h, SD 37.68 vs. 46.72, SD 48.55, respectively; *P* = 0.11).

The perioperative mean and median blood loss were greater in patients receiving DOACs than in patients receiving antiplatelet agents or VKAs (Table 5).

**Table 5:** Perioperative blood loss, major bleeding and mortality at 90 days stratified by the type of antithrombotic drug used

	None	Antiplatelet agents	Vitamin K antagonists	Direct oral anticoagulants	*P*
Blood loss cc (mean and SD)	1195 (666)	1322 (710)	1227 (589)	1438 (738)	0.008
Blood loss cc (median; IQR)	1109 (696–1554)	1247 (857–1689)			0.076
Blood loss cc (median; IQR)	1109 (696–1554)		1043 (864–1652)		0.504
Blood loss cc (median; IQR)	1109 (696–1554)			1294 (937–1824)	0.002
Major bleeding	2.4%	3.0%	2.7%	4.2%	n.s.
*Type of major bleeding*					
Haematuria	3	1	1	2	
Gastrointestinal	1	2	0	1	
Haematoma of thigh	2	4	1	1	
Haemorrhagic stroke	0	0	0	1	
Mortality at 90 days	6.6%	8.9%	10.6%	7.4%	n.s.

*IQR* interquartile range

The rates of major bleeding and mortality at 90 days were similar across subgroups of patients using different types of antithrombotic drug (Table 5).

Perioperative blood loss, major bleeding and mortality were similar in patients on DOACs with or without preoperative DOAC measurements (Table 6).

**Table 6:** Blood loss, major bleeding and mortality at 90 days with and without preoperative DOAC measurements

	Preoperative DOAC measurement (*n*: 34)	Without preoperative DOAC measurement (*n*: 114)	*P*
Blood loss cc (mean and SD)	1478.1 (768.5)	1424.85 (731.8)	n.s
Blood loss cc (median, IQR)	1468 (855–1939)	1285 (952–1779)	
Major bleeding	9.6%	2.8%	n.s
Mortality at 90 days	5.8%	9.6%	n.s

Supplemental Table 1 shows the DOAC levels measured in the morning after admission and in the morning after surgery according to the different types of drugs used. On the morning of surgery, DOAC levels were close to or lower than the through levels reported in the literature for all types of DOACs [33]. Supplemental Table 2 shows the characteristics of patients who underwent surgery within 48 h compared with those who underwent delayed surgery (> 48 h).

Table 7 shows the univariable and multivariable logistic regression of the baseline characteristics of patients and the DOAC assumption in relation to the timing of surgery (< 48 vs. > 48 h). The CIRS-C comorbidity score, low haemoglobin at admission, and DOAC use were significantly associated with surgical delay longer than 48 h, whereas female sex and the centre of Bologna were associated with a surgical delay of less than 48 h.

**Table 7:** Univariate and multivariate logistic regression analyses of characteristics associated with delayed surgery (> 48 h)

	Univariate OR (95% CI)	*P*	Multivariate OR (95% CI)	*P*
Gender (female)	0.59 (0.42–0.84)	0.00	0.63 (0.40–0.99)	0.05
Age	1.005 (0.98–1.03)	0.72		
CIRS-S	1.24 (0.87–1.76)	0.22		
CIRS-C	1.23 (1.11–1.37)	0.00	1.13 (1.01–1.26)	0.03
ASA score	1.10 (0.77–1.56)	0.61		
ADL	0.98 (0.90–1.08)	0.76		
Barthel Index	1.00 (0.99–1.00)	0.94		
SPMSQ	0.99 (0.94–1.06)	0.96		
Haemoglobin at admission gr	0.90 (0.82–0.99)	0.03	0.86 (0.76–0.96)	0.01
Albumin gr	0.79 (0.50–1.25)	0.31		
Type of fracture (% intracapsular)	0.908 (0.63–1.31)	0.61		
DOACs (%)	2.96 (2.02–4.34)	0.00	2.48 (1.55–3.96)	0.00
Centre (Modena vs. Bologna)	1.36 (1.16–1.61)	0.00		
Centre (S. Giovanni Rotondo vs. Bologna)	2.49 (1.33–4.67)	0.00		
Centre (all vs. Bologna)	1.37 (1.16–1.61)	0.00	1.33 (1.07–1.66)	0.01

ADL, Activity of Daily Living Katz Index; ASA, American Society of Anaesthesiology; IQR, interquartile range; CIRS, Cumulative Illness Rating Scale; CIRS-S, Cumulative Illness Rating Scale- Severity Index; CIRS-C, Cumulative Illness Rating Scale Comorbidity index; SPMSQ, Short Portable Mental Status Questionnaire

## Discussion

No previous studies have reported changes in the duration of perioperative DOAC interruption or perioperative blood loss based on residual anticoagulant levels.

Our results indicate that DOAC level testing may delay hip fracture surgery without reducing total blood loss, major bleeding or mortality at 90 days. Patients receiving DOACs had significantly greater total blood loss than did those not receiving antithrombotic agents, even amongst subjects with DOAC low residual presurgical levels, but there was no effect on mortality. These findings support the development of guidelines regarding the timing of surgery in patients on DOACs without the need to measure residual anticoagulant activity.

Delayed surgical repair is associated with worse outcomes in elderly patients with hip fractures. However, a relevant proportion of these patients are also administered oral antithrombotic agents (either antiplatelet agents or anticoagulants) [13]. Therefore, the effects of these drugs should be rapidly reversed before surgery to reduce the risk of bleeding.

DOACs have replaced VKAs as the anticoagulants of choice for treating AF and venous thromboembolism. The effect of VKAs can be measured by the INR, and they use vitamin K as a reversal agent; however, at least 12 h are needed to exert an effect. Reversal agents are also available for DOACs, but they are only approved for life-threatening bleeding in the case of andexanet alfa, which can reverse the factor Xa inhibitors apixaban and rivaroxaban. Idarucizumab is available for dabigatran reversal in the case of urgent surgery. However, the cost, availability, preparation, and risk of thrombosis may complicate the use of these reversal agents. DOACs have a short half-life, and international guidelines suggest that surgery at a high risk of bleeding should be delayed at least 48 h after the last dose of apixaban, rivaroxaban or edoxaban and up to 96 h after the last dose of dabigatran without measuring DOAC plasma levels [23, 24]. However, these guidelines are not specific to hip fracture surgery. In the case of time-sensitive surgery, guidelines suggest that surgery should only be delayed 24 h after the last DOAC dose.

A recent systematic review and meta-analysis evaluated the effects of expedited surgery protocols on the time to surgery and perioperative outcomes in anticoagulant-treated patients with hip fracture [26]. This review included 14 studies, 12 of which were retrospective and only two were prospective cohort studies. Only six studies were eligible for meta-analysis, and the quality of the included studies varied. The mean time to surgery for VKA-treated patients was significantly shorter in those in whom a VKA-reversal protocol was applied [26]. No studies involving DOAC-treated patients have compared the time to surgery before and after an expedited surgery protocol. In the three studies in which the authors reported the mean time to surgery for DOAC-treated patients who underwent expedited surgery and compared them to that of control patients, the mean time to surgery was 29.3 h and 30.2 h, respectively.

Our population included older subjects with a median age of 85 years and a high comorbidity and severity index who were enrolled in three orthopaedics units in Italy. The 90-day mortality rate was determined based on data from the National Outcome Registry (PNE) [3].

Some limitations should be acknowledged. Our study had a retrospective design, and the number of subjects receiving DOACs and DOAC measurement was limited, thus limiting the statistical power to detect a difference in time to surgery between patients who underwent testing and those who did not undergo testing. The relatively long study period may have led to secular changes in the study populations and care practises over time as historical controls were considered.

DOAC level measurements were performed only from 2015 to 2018, when the frequency of patients receiving DOACs increased and guidance on preoperative DOAC testing was limited. Preoperative anticoagulant activity measurements were performed in all patients taking DOACs in Bologna, where all patients underwent neuraxial anaesthesia. After 2018, the number of patients receiving DOACs increased with potential further delays in the timing of surgery, and preoperative DOAC measurements were stopped to allow expedited surgery. In addition, major bleeding events were adjudicated locally and not centrally, whereas perioperative blood loss was calculated centrally. Three different orthopaedic units were involved, and different surgical strategies to limit blood loss could have been employed, such as efficient surgical fixation, topical application of haemostatic agents, and intraoperative cell salvage. Different anaesthesiological interventions to limit blood loss could also be employed (e.g. positioning, regional anaesthesia, permissive hypotension, avoidance of hypothermia, judicious administration of blood products, and the use of systemic haemostatic agents such as tranexamic acid) [36].

In conclusion, our results indicate that DOACs and DOAC level testing may delay hip fracture surgery without reducing blood loss, major bleeding or mortality, consistent with previous findings [37].

Further studies are warranted to develop expedited surgical protocols for hip fracture patients receiving DOACs.

## Supplementary Information

Below is the link to the electronic supplementary material.

Supplementary file1 (DOCX 52 KB)

## Data Availability

The datasets used and analysed during the current study are available from the corresponding author upon reasonable request.
