# Supplementary material for: A comparison of the effects of direct oral anticoagulants versus vitamin K antagonists and antiplatelet agents on the timing and outcomes of hip fracture surgery in patients older than 65 years: the ORTHO-GER-DOAC study
Source: Eur Geriatr Med. 2025 Apr 23;16(3):899–907. doi: 10.1007/s41999-025-01198-9 (PMC12174301; doi:10.1007/s41999-025-01198-9)
Supplement: Supplementary file 1 — Supplementary file1 (DOCX 52 KB) [file 41999_2025_1198_MOESM1_ESM.docx]

Supplementary Table 1: Preoperative direct oral anticoagulant (DOAC) measurements

| Patients on different types of DOACs  (number; %) | Number of patients undergoing preoperative DOAC measurement | DOAC levels  (morning after admission)  mean (SD) ng/mL  median (IQR) ng/mL | DOAC levels (morning of surgery)  mean (SD) ng/mL  median (IQR) ng/mL |
| --- | --- | --- | --- |
| Apixaban (80; 54) | 22 | 121.1 (89.5)  95.5 (52.9-165) | 47.88 (32)  42.00 (29.5-62.25) |
| Rivaroxaban (34;23) | 6 | 29.3 (24.3)  28.0 (6-48) | 8.67 (10.2)  6.0 (0-20) |
| Dabigatran (19; 13) | 4 | 101.5 (107.2)  70 (20-214.5) | 45.5 (0.7)  45.5 (40-49) |
| Edoxaban (15; 10) | 2 | 24.8 (5.4)  24.85 (21-24) | 17.0 (6.6)  17.0 (17-17) |

SD: standard deviation

Supplemental Table 2: Characteristics of subjects who underwent surgery within 48 hours compared with those who underwent delayed surgery (> 48 hours)

|  | Surgery  within 48 hours | Surgery  after 48 hours | p |
| --- | --- | --- | --- |
| Gender (% Female) | 77.9% | 67.6% | 0.03 |
| Age median IQR | 85 (80-89.25) | 86 (81.25-89) | 0.43 |
| CIRS-S median IQR | 1.76 (1.38-2.0) | 1.84 (1.53-2.09) | 0.03 |
| CIRS-C median IQR | 2 (1-4) | 3 (2-5) | 0.00 |
| ASA score median IQR | 3 (3-3) | 3 (3-3) | 0.37 |
| ADL median IQR | 5 (3-6) | 5 (3-6) | 0.53 |
| Barthel Index median IQR | 90 (60-100) | 90 (63-100) | 0.89 |
| SPMSQ median IQR | 3 (1-6) | 3 (1-6) | 0.83 |
| Haemoglobin at admission gr%  median IQR | 12.2 (11.1-13.2) | 11.85 (10.7-13.1) | 0.02 |
| Albumin gr median IQR | 3.4 (3.2-3.7) | 3.4 (3.0-3.7) | 0.54 |
| Type of fracture (% Intracapsular) | 42.2 | 39.8 | 0.61 |
| DOACs (%) | 13.5 | 31.7 | 0.00 |

ADL: Activity of Daily Living Katz Index; ASA: American Society of Anaesthesiology; IQR: interquartile range; CIRS: Cumulative Illness Rating Scale; CIRS-S: Cumulative Illness Rating Scale- Severity Index; CIRS-C: Cumulative Illness Rating Scale Comorbidity index; SPMSQ: Short Portable Mental Status Questionnaire
